# The lingering crisis: gaps in long-term care for children with congenital Zika syndrome and their families in Brazil

**DOI:** 10.1590/S1678-9946202567036

**Published:** 2025-06-09

**Authors:** Paulo Ricardo Martins-Filho

**Affiliations:** 1 Universidade Federal de Sergipe, Hospital Universitário, Laboratório de Patologia Investigativa, Aracaju, Sergipe, Brazil

Aracaju, May 1^st^, 2025

Dear Editor,

Recently, an international consortium of experts proposed a renewed research and development agenda for Zika virus preparedness, warning that the decline in political commitment and global investment following the epidemic has left critical gaps in surveillance, diagnostics, and long-term care systems^1^. Since the 2015–2016 outbreak, more than 1,850 children were born with confirmed congenital Zika syndrome (CZS) in Brazil and the absence of sustained strategic planning has compromised the continuity of essential services. As these children grow, their complex clinical and developmental needs demand continuous access to high-quality, multidisciplinary rehabilitation.

Within the Brazilian Unified Health System (SUS), the progressive reduction and fragmentation of specialized care programs has disrupted service delivery, leading to delays in treatment, doubled efforts, and missed opportunities for early intervention^2^. These gaps also imposed financial burdens on families and deepened existing social inequalities, particularly among those in vulnerable socioeconomic conditions^3^. Recent studies^4^ have demonstrated that over 90% of families affected by severe CZS report catastrophic health expenditures—often exceeding 40% of household income—with major costs arising from transportation, medication, nutritional formulas, medical supplies, home adaptations, and caregiving services.

Additionally, there was a decline in the provision of social security benefits to families affected by CZS after the Zika virus epidemic, without any scientific or social justification—revealing the absence of a structured institutional response to uphold the rights of children with microcephaly^5^. Complementary data^6^ from a cohort in Rio de Janeiro indicate that although 65% of families eventually accessed the federal government's benefit, nearly half experienced delays as long as six months, while others discontinued the application process due to bureaucratic obstacles, emotional exhaustion, or lack of guidance. These institutional shortcomings not only undermine access to basic rights, but also disrupt family functioning, leading to shifts in occupational roles and a significant reduction in overall income-generating capacity.

Within families affected by CZS, mothers most often assume caregiving responsibilities, whose withdrawal from wage employment has been strongly associated with psychological distress. Studies report a seven-fold increase in the likelihood of common mental disorders among women who left the workplace to provide full-time care^7^. This burden is intensified for single mothers, who face additional challenges due to the absence of shared caregiving support and greater exposure to economic vulnerability^8^. Combined with limited institutional support, these overlapping pressures have led some families to abandon efforts to access social protection mechanisms—what researchers^6^ describe as a "context of disincentive and discouragement." These findings indicate the need for integrated strategies that support caregivers’ mental health, reduce the cumulative burden on families, and promote pathways for occupational reintegration.

Geographical disparities further complicate these challenges, especially in remote or underserved areas^9^. Studies investigating access to specialized health services in Northeast Brazil found that families often travel long distances to reach care facilities, facing logistical, time, and financial barriers^2,10^. These structural issues became more evident during the COVID-19 pandemic, which intensified participation restrictions for children with CZS and limited their involvement in daily routines, therapy sessions, and education—further isolating them from support networks^11^.

Moreover, qualitative research^12^ with caregivers has identified multiple environmental and systemic barriers to social participation and continuity of care, including ableism, inaccessible infrastructure, inadequate provision of assistive technologies, and lack of standardized care protocols. Despite legal provisions for integrated care, services remain fragmented and poorly adapted to the needs of children with CZS, especially in terms of food preparation, transportation, and access to rehabilitation services. These findings emphasize the need for localized, family-centered approaches.

Despite these systemic obstacles, evidence supports that rehabilitation can significantly improve outcomes for children with CZS. Neurodevelopmental therapies have been shown to enhance motor function and reduce the incidence of secondary infections such as pneumonia and urinary tract infections^13^. Sustained, individualized care not only improves child health but also alleviates caregiver burden, enhances quality of life, and may prevent institutionalization. Early and continuous intervention fosters inclusion, education, and long-term autonomy. A combination of targeted system-level strategies is essential to address persistent risks and promote sustainable outcomes for children and families affected by CZS:

- Policy strengthening: expand national policies ensuring long-term funding for multidisciplinary CZS care, with dedicated budgets and performance indicators^2,5^.
- Integrated care networks: link primary, secondary, and tertiary services through referral protocols, shared records, and case management teams to ensure continuity of care^14^.
- Community-based services: expand telehealth and train local providers to reduce travel burdens and improve culturally sensitive access in rural areas^15^.
- Caregiver support: offer psychoeducational programs, peer support groups, respite care, and financial assistance to strengthen caregiver resilience^16,17^.

Brazil's initial response to the Zika virus epidemic demonstrated operational capacity and intersectoral coordination under emergency conditions. However, the absence of a sustained institutional framework has since compromised equitable and continuous care for children with CZS. Transitioning from short-term emergency actions to permanent, systematized policies is essential to prevent avoidable morbidities and mitigate structural inefficiencies. This requires the implementation of robust rehabilitation models within the SUS, grounded in stable funding mechanisms, interprofessional coordination, and routine monitoring of outcomes. Investing in long-term rehabilitation is not only clinically and socially necessary—it is a strategic use of public resources that can reduce reliance on high-cost tertiary care.
